# P325: Evaluation of rational prescribing of essential generic drugs in a rural community in Mali

**DOI:** 10.1186/2047-2994-2-S1-P325

**Published:** 2013-06-20

**Authors:** M Sanogo, S Maiga, B Koumare, OS Maiga

**Affiliations:** 1 Faculty of Medicine, School of Public Health, University of Montreal, Montreal, Canada; 2 Faculty of Medicine, Pharmacy and Dentistry, Bamako, Mali

## Objectives

To assess the quality of the prescription of generic essential drugs in a reference health center in Mali.

## Methods

This is a descriptive cross-sectional study which was conducted from March to December 2008. The sample consisted of 300 prescriptions in outpatient. The parameters studied were the reasons for consultation, diagnosis retained, prescription drugs, and information on training for prescribers, standards and the treatment regimen.

## Results

There were 1036 drugs prescribed for 300 prescriptions. Drugs most prescribed were anti-infectives, then analgesics, antipyretics and antimalarials. The average number of drugs per prescription was 3.4. In 70 % of prescriptions generics were prescribed, doctors were prescribers for only 64% of prescriptions. The average number of brand-name medicines per prescription was 1.03. The percentage of orders that contained at least one brand-name medicine was 62.33 %. Influencing factors evoked by prescribers were: efficiency for 57.9 %, the cost (31.6 %) and the availability (10.5 %). The mean cost of the order was 4400 CFA francs (about seven euros).

## Conclusion

Brand names continue to be widely prescribed in the rural town of San. A medicine prescription of international nonproprietary names is highly recommended.

## Disclosure of interest

None declared

